# Effect of improved cookstove intervention on childhood acute lower respiratory infection in Northwest Ethiopia: a cluster-randomized controlled trial

**DOI:** 10.1186/s12887-020-02459-1

**Published:** 2021-01-04

**Authors:** Mesafint Molla Adane, Getu Degu Alene, Seid Tiku Mereta, Kristina L. Wanyonyi

**Affiliations:** 1 Department of Environmental Health, College of Medicine & Health Sciences, Health, School of Public Health, Bahir Dar University, Bahir Dar, Ethiopia; 2 Department of Epidemiology and Biostatistics, College of Medicine & Health Sciences, School of Public Health, Bahir Dar University, Bahir Dar, Ethiopia; 3 Department of Environmental Health Sciences and Technology, Jimma University, Jimma, Ethiopia; 4 Institute of Dentistry, Barts and The London School of Medicine and Dentistry, Queen Mary University of London, London, UK

**Keywords:** Acute lower respiratory infection, Biomass-fuelled, Childhood, Household air pollution, Improved cookstove

## Abstract

**Background:**

Household air pollution exposure is linked with over 3.5 million premature deaths every year, ranking highest among environmental risk factors globally. Children are uniquely vulnerable and sensitive to the damaging health effects of household air pollution which includes childhood acute lower respiratory infection (ALRI). The use of improved cookstoves has been widely encouraged to reduce these health burdens. It is, however, unclear as to whether it is possible to prevent household air pollution-related disease burdens with biomass-fuelled improved cookstove intervention and the evidence regarding its child health effect still attracts wide debate. Therefore, we investigated the child health effect of improved baking stove intervention compared with the continuation of the open burning traditional baking stove.

**Methods:**

A cluster-randomized controlled trial was conducted to assess the health effect of improved baking stove intervention. A total of 100 clusters were randomly allocated to both arms at a 1:1 ratio, and a total of four follow-up visits were carried out within 1 year immediately after the delivery of the intervention to all households allocated into the intervention arm. Data were analyzed in SPSS-22, and the intervention effect was estimated using a Generalized Estimating Equations modeling approach among the intention-to-treat population.

**Results:**

A total of 5508 children were enrolled in the study across 100 randomly selected clusters in both arms, among which data were obtained from a total of 5333 participants for at least one follow-up visit which establishes the intention-to-treat population dataset. The intervention was not found to have a statistically significant effect on the longitudinal childhood ALRI with an estimated odds ratio of 0.95 (95% CI: 0.89–1.02). Nevertheless, the longitudinal change in childhood ALRI was significantly associated with age, baseline childhood ALRI, location of cooking quarter, secondary stove type and frequency of baking event measured at baseline.

**Conclusions:**

We found no evidence that an intervention comprising biomass-fuelled improved baking stove reduced the risk of childhood ALRI compared with the continuation of an open burning traditional baking stove. Therefore, effective cooking solutions are needed to avert the adverse health effect of household air pollution, particularly, childhood ALRI.

**Trial registration:**

The trial was registered on August 2, 2018 at clinical trials.gov registry database (registration identifier number: NCT03612362).

## Background

Household air pollution (HAP) exposure from cooking with solid fuels is linked with over 3.5 million premature deaths every year; including half a million deaths among children under the age of 5 years from pneumonia worldwide, and it is ranked highest among the environmental risk factors globally [1]; predominantly, in low-and-middle-income countries (LMICs) [2]. Its burden is very high in the Sub-Saharan Africa region due to the widespread use of polluting household cooking technologies [3]. Although HAP affects all stages of life [4], children are uniquely vulnerable and sensitive to the damaging health effects of HAP [5] due to both their physiology as well as to the extent of their exposure [6]. There is substantial evidence that HAP exposure from solid-fuel use increases the risk of childhood respiratory infections [7] such as childhood acute lower respiratory infection (ALRI) [8].

The improved cookstove (ICS) intervention is a common practice to reduce the burden of HAP-related diseases [4]. It is, however, unclear as to whether it is possible to prevent HAP-related disease burdens with biomass-fuelled ICSs [9], and the evidence on the health effect of ICS interventions is still open to questions. In particular, the evidence regarding the effect of biomass-fuelled ICS intervention on childhood ALRI is still debatable. For example, a study based on a field experiment in Guatemala reported that a 63.3% reduction in respiratory symptoms among children following wood-fuelled ICS intervention [10], and another randomized controlled trial reported a significant risk reduction in childhood pneumonia following ICS intervention with a rate ratio of 0·82 (95% CI: 0·70, 0·98) among intervention group in Guatemala [11]. In contrast, based on experimental evidence, a recent policy research working paper reported no evidence that “Mirt” IBS intervention reduced the risk of respiratory symptoms among older children in rural Ethiopia [12]. Correspondingly, a recent ICS trial found no evidence that biomass-fuelled ICS intervention reduced the risk of pneumonia among children in rural Malawi with an incidence rate ratio of 1·01 (95% CI:0·91, 1·13) [13]. In general, it becomes complex to burn biomass fuel cleanly in household appliances [14], and several recent systematic reviews concluded that evidence concerning the health effect of biomass-fuelled ICS interventions in LMICs is still sparse, heterogeneous, and inconclusive [13, 15, 16].

In Ethiopia, despite some pilot trial efforts on HAP and respiratory symptoms reduction [12, 17] and energy efficiency tests [18], trial evidence on the health impact of ICS intervention is negligible, and recent systematic reviews on this subject matter reported that such type of evidence is lacking and should be a research priority area in Ethiopia [18, 19]. In addition, the ICS trial evidence from other LMICs [11, 13] cannot be directly generalized to the Ethiopian perspective from a relevance point of view, such as the particular variation of cooking device requirements and trial stove designs [20]. Beyond the debatable biomass-fuelled ICS trial reports worldwide [11, 13], little attention has been paid so far to the health effect of biomass-fuelled ICS in Ethiopia.

Hence, the health effect of the biomass-fuelled *“Mirt”* improved baking stove (IBS) intervention, is of keen interest, as it is a well-known commercially distributed type of IBS in Ethiopia [21, 22]. Research into the health effect of the “Mirt” stove had not been satisfactorily dealt with in earlier studies [12] emphasizing the need for further investigation on most vulnerable populations such as children who are often exposed to this intervention. More precisely, a crucial question whether the Ethiopian IBS intervention leads to a decrease in the occurrence of childhood acute lower respiratory infection (ALRI) must be answered through further trial investigation as recommended by a previous study [23]. Thus, given the high magnitude of HAP problems in Ethiopia [19, 24, 25] and lack of IBS trials [18, 19], there is a need to test the health effect of IBS intervention that could potentially reduce the negative health impacts of HAP.

Because of that, we conducted a community-level cluster randomized controlled trial to assess the effect of biomass-fuelled IBS intervention on childhood ALRI at the individual child level in Northwest Ethiopia compared with the continuation of an open burning traditional baking stove (TBS). The major rationale for adopting a cluster-randomization technique was to prevent contamination [26] or unintentional spill-over of intervention effects from one treatment group to another of the trial if individual household randomization was used, as children would inevitably be exposed to the risk or intervention in neighbor households. The other rationales were to increase administrative effectiveness, minimize costs, and to eliminate potential ethical problems [27].

The benefits of the study go beyond addressing an important research gap. The benefits of this study can be grouped into three categories; population health, policy, and economical benefits. When considering population health, since the primary outcome sought is a reduction in childhood ALRI from an effective biomass smoke exposure reduction intervention, it would achieve a high health impact on most vulnerable children living in developing countries like Ethiopia. The study would also achieve a high policy and operational benefits by generating new evidence needed by local, national and international policy-makers and donors regarding health effects seen when households adopt IBS technology as well as it can also inform governments in LMICs how to allocate the limited resources to focus their efforts on the most effective strategies to improve health.

## Methods

### Study locations and context

Ethiopia is located in the Northeastern part of Africa, known as the Horn of Africa, and occupies an area of 1.1 million square kilometers ranging from 4620 m above sea level at Ras Dashen Mountain to 148 m below sea level at the Danakil depression [28]. It possesses three major topographic-induced climatic zones, the hot lowlands (“*Kolla*”) located below 1500, the temperate (“*Wayna Dega*”) which range 1500–2400, and the cool temperate highlands (“*Dega*”) located above 2400 m above sea level [28, 29]. The mean annual temperature is around 15–20 °C and 25–30 °C for highlands and lowlands respectively [29].

This trial was conducted in a low-income rural community of the Mecha Health and Demographic Surveillance System (MHDSS) site. MHDSS site is a field research center established in 2013 by Bahir Dar University to conduct and support postgraduate level studies in the region. It is located 525 km away from the capital city of Ethiopia, Addis Ababa, towards Northwest and 40 km far away from the capital city of Amhara Regional State, Bahir Dar. According to the official population profile report of MHDSS, the study area comprises 132 clusters/ “*Gots*” with a total of 65, 086 populations within 20,631 households at the end of 2016. Out of which children less than 4 years old were accounted for 13.3% of the total population. Biomass-fuel burning in open traditional cookstove was the major (94.5%) household energy source in the study locality [23, 30].

### Trial design

With a longitudinal experimental design, a community-level cluster randomized controlled trial study with two arms of equal size was used to compare the effect of biomass-fuelled IBS intervention with TBS on childhood ALRI at an individual child level. Clusters were the small villages, termed as “*Gots*” in Amharic (both national & local language), are the lowest administrative units in Ethiopia, and used as the smallest unit of enumeration areas by the Ethiopian national census authority. Each cluster (“*Got*”) was comprised about 55 eligible children on average, and all eligible children in the selected clusters were enrolled as control or intervention for baseline and repeated follow-up visits approximately every 3 months for 1 year after receiving the intervention. With this design, childhood ALRI outcome was measured before installation of IBS, and again in the same households, 4 times after the intervention households received the IBS. The households with TBS method were served as a control arm.

### Eligibility criteria

All clusters/ “*Gots*” under MHDSS site were eligible for participation in the trial; and to ensure a minimum of 1 year repeated visit for longitudinal data collection before the child’s 5th birthday, all households with at least one less than 4 years old child and who were exclusive users of TBS were eligible for participation in the trial. Merely households who did not have any enclosed baking quarter (kitchen) structure and children who were born during the course of the study were excluded.

### Sample size determination

To estimate the effect of IBS intervention on child ALRI risk reduction over 1 year follow-up period, the sample size was calculated by applying the two-sample comparison of proportions formula using STATA. We calculated the required sample size by assuming childhood ALRI prevalence of 21% from a previous study in rural Ethiopia [31] and 0.05 two-tailed alpha to detect a 25% reduction in the prevalence of childhood ALRI with a power of 80%. Accordingly, the estimated sample size (n_1_) was 891 children per arm with equal numbers to both arms under individual randomization.

However, since this trial was randomized the intervention over clusters instead of individual households, the sample size was calculated using the standard formulae for unequal size cluster randomized controlled trials as:

n_c_ = n_1_ [1 + (m ’  − 1) ICC] [32].

Where, n_c_ = sample size with cluster randomization, n1 = sample size under individual randomization, m’ = an average number of eligible children within each cluster, and ICC = intra-cluster correlation coefficient for cluster level ICC of childhood ALRI.

Thus, the required sample size (n) was 2068 per arm under equal allocation by assuming an ICC value of 0.03; which is an estimated value for child pneumonia outcome in the cookstove intervention study [13], and an average number of eligible children of 55 within each cluster from the updated data of MHDSS.

The number of clusters (K) required in each arm for unequal cluster sizes was also determined using the formula:

K = n [1 + ((CoV^2^ + 1) m ’  − 1) ICC]/m ’ ] [32].

Where, CoV = coefficient of variation.

The number of clusters per arm became 40, and this was caused to increase the sample size to 4400 (2200 per arm) by considering an effective ICC value of 0.03 for cluster-level ICC value of childhood ALRI, a CoV value of 0.3 for cluster size and an average number of eligible children of 55 within each cluster.

As a final point, considering the nature of cluster sampling method, we added 25% of the sample to account for lost to follow-up (LTF) and any other unpredictable events in the field [33, 34], the required sample size (n) was increased to about 2750 within 50 clusters per arm, which would provide a total of 100 clusters containing about 5500 eligible children randomized in equal numbers to both arms.

### Randomization and masking

Clusters were randomly allocated to intervention and control arms at a 1:1 ratio by an independent epidemiologist using a computer-generated randomization schedule, which was revealed after all baseline measurements had been completed as well as all study households recruited and assigned to their respective arm to ensure the allocation sequence was concealed from those assigning the arms. Both study participating households and data collectors were blinded to intervention status during study enrollment and baseline data collection. In addition, all eligible children/households within the clusters were included in the study to minimize the risk of selection bias; however, because of the distinctive feature of cluster design and nature of the intervention under study, blinding of the households receiving the IBS intervention was not possible.

### Sampling method and recruitment of participants

The cluster sampling method was used to randomly select 100 clusters (50 clusters per arm) among the total 132 clusters in the MHDSS site, and all eligible households were included within the selected cluster (complete enumeration). The sampling frame, the list of clusters and the potential eligible households with less than 4 years old child, was established from the MHDSS record. Then, the eligible households were chosen from the record and a child less than 4 years old was recruited from each household. In situations where there were two or more children less than 4 years old living in the same household, only the youngest child was included in the study.

The selected households were identified using the permanent MHDSS site house number and the actual participants were recruited at the household level by field workers during the baseline survey after ensuring whether the households met the eligibility criteria. A screening questionnaire was used by field data collectors upon their first visit to each household to ensure that the household was appropriate and willing to participate. When the household meets the eligibility criteria, the study was explained to the index child parents, and they were asked whether the household is willing to participate in the study and use improved baking stove technology for at least 12 months.

Then, when the parents of the household agreed to be involved in the study, the field staff administered a written consent form at that time and the consent procedure was conducted in Amharic (both national & local language). It was also explained that the allocation to intervention or the control group was based on the concept of a “lottery” method, and several special efforts were applied during recruitment to facilitate the enrolment process as described within our earlier research report [23], and there was no household unwilling to participate in the study.

### Intervention

#### Trial descriptions

Replacing the open burning TBS with *“Mirt”* IBS, the well-known commercially distributed type of IBS in Ethiopia, was the intervention for this study. *The “Mirt”* IBS intervention can save a considerable quantity of fuel-wood compared to the TBS method [35], and it is a biomass-fuelled without chimney stove designed by the Ethiopian Energy Studies Research Center for cooking the staple food of Ethiopia called “*Injera*” (i.e., a unique type of yeast-risen flatbread, consumed widely in Ethiopia) [21, 22].

All households who were randomized to the intervention arm were identified using the permanent MHDSS house number for a convenient appointment date, and the intervention was delivered to all eligible households in 50 randomly allocated clusters at the beginning of the study period, and the control households were continued to use the customary open burning TBS method equally in 50 randomly allocated clusters. All the trial cookstoves were manufactured by a local licensed firm and installed on-site by the installation teams.

Demonstration in the use of the IBS was also provided to each household during the time of installation, and the IBS intervention was promoted regularly throughout the follow-up period by the local energy experts’ team of IBS monitors. Concerning study duration, since the life span of “*Mirt”* IBS is about 5 years [21, 22], the length of both the intervention and the follow-up period was 1 year safely to account for seasonal factors that have a major effect on the magnitude of HAP in Ethiopia [36], and to maintain a sufficiently short follow-up period to decrease attrition.

#### Trial adherence and compliance monitoring

Participants’ adherence to the intervention assigned to them were assessed through self-report and direct observation by trained field enumerators along with local energy experts’ team in both arms. At each follow-up visit, the enumerators observed and recorded the type and condition of baking stove currently being used (i.e., no observed breakage resulting in no use). Additionally, the primary cook was asked whether the baking stove intervention was in good working order (i.e., no reported breakage resulting in no use).

Accordingly, timely response to trial-related difficulties such as rapid maintenance or replacement of defective baking stove were accomplished by the installation teams as needed to improve intervention protocol adherence and avoid the potential detrimental effects of non-adherence. In addition, trial protocol compliance was checked by the local energy experts’ team of stove monitors’ through unannounced visual inspection visits in homes of both arms to enhance data validity.

#### Participant retention strategies

Once the households were enrolled, reasonable efforts were made to promote participant retention and complete follow-up for the entire study period by working on active community engagement through the Ethiopian health extension program as well as through the local health development army team structure to prevent missing data and avoid the associated complexities in analysis and interpretation. In this regard, interest in the study was maintained through periodic communications about the intervention protocol adherence during the regular local health development army team meetings as well as during home visits by field workers, health extension workers, and local energy experts.

Furthermore, home visits were scheduled to limit the participants’ burden related to follow-up visits, and at the start of the trial, control households were informed that they would receive the IBS intervention at the end of the study period to maintain justice and achieve a high level of post-recruitment participant retention.

#### Trial safety monitoring

Even though the *“Mirt”* IBS intervention [21, 22], which was tested by this trial, was not involved any drug or medical procedure; and not known to increase the risk of any adverse event, masked interim analyses were included in the protocol for safety and efficacy monitoring. Since the standard *“Mirt”* IBS intervention [21, 22] is expected to reduce HAP related health effects [22], it was likely to be safer than the open burning TBS method. Any adverse events data deemed related to the trial intervention (i.e., such as cooking-related burn events data) were collected, and reported immediately for appropriate medical action and for further assessment of seriousness and expectedness to inform the conduct of the ongoing trial.

The collected data were reviewed for safety in December 2018 by an independent data Safety, and Monitoring Board to determine whether there were grounds to stop the trial for adverse events. Nevertheless, the Board found no grounds to stop the trial early due to adverse events.

### Outcome assessment

To identify the occurrence of childhood ALRI, we used the definition of Integrated Management of Childhood Illnesses (IMCI) pneumonia algorithm developed by the World Health Organization (WHO) [37, 38]. Since nurses can effectively diagnose childhood pneumonia, and the IMCI-pneumonia assessment protocol is a common diagnostic criterion at health facilities in Ethiopia [39], the outcome variable was assessed in the same manner in both arms by field nurses, who were trained in IMCI-pneumonia algorithm, and not involved in either randomization or intervention delivery. In this trial, the term childhood ALRI was used as a synonym for the IMCI-pneumonia [37, 38], because it is the preferred term for childhood pneumonia in peculiar to the developing countries [40].

### Data collection methods

Baseline data were collected in each household before intervention delivery, and a total of four follow-up visits were carried out, immediately after the delivery of the intervention, in the same week at approximately 3-month intervals for longitudinal data collection by trained local field nurses. The duration of the follow-up period was 1 year to cover the major Ethiopian periods of annual weather changes and to account for seasonal factors that might have a major effect on the magnitude of both ALRI and HAP in Ethiopia [41].

### Data quality assurance

Various appropriate measures were taken to address the validity and reliability of the study by ensuring the quality of data. To start with the outcome variable, childhood ALRI was assessed in the same manner in both arms by field nurses who were trained in the standard IMCI-pneumonia algorithm. The study team was in regular contact with the data collection team with scheduled meetings and additional communications as needed for quality control. About 5% of randomly selected home visits were done in duplicate by supervisors as cross-checking mechanism to ensure the validity of the collected data, and feedback sessions were done on about a three-month basis.

To minimize the risk of bias, clusters were randomly allocated to intervention and control arms, all eligible households within the clusters were included in the study, the allocation sequence was concealed from those assigning participants to groups, primary outcome assessors were blind to the intervention and outcome measurement was performed in the same manner in both arms. In addition, the entire trial stoves were manufactured by a single licensed firm and the same installation teams were administered the intervention in both arms.

Furthermore, all initially randomized participants were analyzed in the groups they were assigned to (i.e., intention-to-treat/ ITT analysis principle). Furthermore, the methodological soundness such as large sample size, longitudinal study design, and baseline data collection on the primary outcome and risk factors to be adjusted through GEE modeling can help us to achieve an effective balance of confounders in both arms. Finally, this manuscript was reported following both the guidelines of Consolidated Standards of Reporting Trials (CONSORT) 2010 statement extension to cluster randomized trials [42] to address the essential study design components of this trial report; and Template for Intervention Description and Replication (TIDieR) checklist [43] for better reporting of the intervention aspects.

### Statistical analysis methods

Data were analyzed using the Statistical Package for Social Sciences (SPSS), version 22 and all statistical tests were two-sided with *p*-value < 0.05 considered statistically significant. To quantify the magnitude of clustering for childhood ALRI outcome, cluster-level ICC value was calculated using the standard formulae as:

ICC = Var (*U*_*o*_)/**V**ar (*U*_*o*_) + Π^2^/3 [44, 45].

Where, Var (*U*_*o*_) is the random intercept variance, that is, the estimated cluster-level (i.e., level-two) variance component, and Π^2^/3 refers to the individual-level (i.e., level-one) variance component which is fixed to be 3.29 [45]. We took this fixed value of 3.29 for the individual level variance component, as there is no individual-level (level-one) residual for binary (discrete) outcome variable in the multilevel logistic analysis model [44, 45].

Using this formula, the estimated magnitude of cluster-level ICC value for childhood ALRI outcome was found to be not different from zero, which indicates that only well below 1% of the total variability in the chance of acquiring childhood ALRI is explained by the between cluster-level variation. Therefore, we safely treated individual children as the sole unit of analysis and interpretation in determining the effect of IBS intervention on childhood ALRI [44] compared with the continuation of the open burning TBS.

The effect of IBS intervention on the repeated response of childhood ALRI between the two arms was estimated using adjusted Odds Ratios (AORs), with the respective 95% CIs, as measures of effect following a Generalized Estimating Equations (GEE) modeling approach among the ITT population by considering the underlying design of the study and nature of the outcome variable under investigation. The GEE analysis method is the preferred method for longitudinal data analysis due to its computational simplicity and robustness to misspecification of the repeated measures correlation structure [46]. Besides, this analysis technique can take both the village-level clustering of childhood ALRI outcome and the longitudinal sampling method into account, and it can handle missing data values without the need for explicit imputation by considering all participants with at least one follow-up visit through automatically excluding the missing values by the GEE analysis model [46].

Concerning model fitness, even though the GEE method is understood to be robust against a wrong choice of working correlation structure (WCS), the best WCS of the outcome variable was chosen as autoregressive (AR1) by means of a critical examination of the working correlation matrix of the observed correlations between subsequent measurements [46] to uphold the goodness of model fitness, and hence to get a more precise estimation of the IBS intervention effect.

As a final point, our GEE analysis model has included a binary outcome variable of repeatedly measured childhood ALRI and a binary indicator of treatment allocation (i.e., control versus intervention) as well as other indicator variables measured at baseline such as gender, age, childhood ALRI, location of cooking quarter, secondary cookstove type used for other cooking/boiling water purposes and frequency of “Injera” baking event; and the results are presented next in texts and tables.

## Results

### Baseline characteristics of enrolled participants

**Fig. 1:**
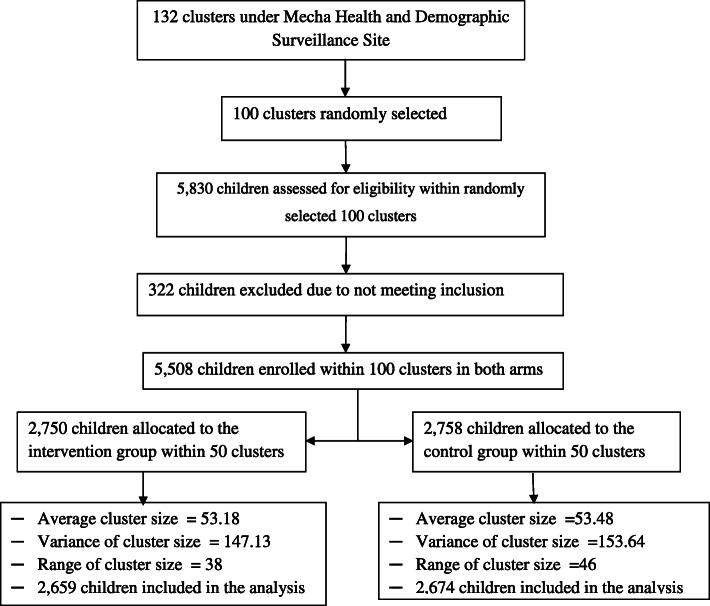
Flow diagram of study progress from eligibility assessment to enrollment, follow-up, and analysis for the trial study entitled effect of improved cookstove intervention on childhood acute lower respiratory infection in Northwest Ethiopia

Among the total 5830 children assessed for eligibility at baseline survey, 5508 (94.48%) children (i.e. one child in each surveyed household) were enrolled in the study from June to August in 2018 across 100 randomly selected clusters in both arms. The trial was comprised of 2750 (49.9%) and 2758 (50.1%) children in the intervention and control arm respectively, and the baseline prevalence of childhood ALRI among the primarily enrolled children was 19.1% (95% CI:18.1–20.2) with 19.1 and 19.2% in intervention and control arms respectively as displayed in Table 1. A total of 322 (5.52%) children were excluded at baseline due to not meeting the inclusion criteria; and 175 (91 in intervention and 84 in the control arm) children were permanently lost to follow-up (LTF) at the first follow-up visit after initial enrollment (Fig. 1).

**Table 1:** Baseline characteristics of enrolled households and children

Child characteristic		Treatment arm		Total (%)
		Control (%)	Intervention (%)	
Gender of child	Female	1293 (46.9)	1346 (48.9)	2639 (47.9)
	Male	1465 (53.1)	1404 (51.1)	2869 (52.1)
Age of child at entry in years (months)	< 1 Year old (0–11)	764 (27.7)	811 (29.5)	1575 (28.6)
	1 Year old (12–23)	774 (28.1)	748 (27.2)	1522 (27.6)
	2 Years old (24–35)	745 (27.0)	690 (25.1)	1435 (26.1)
	3 Years old (36–47)	475 (17.2)	501 (18.2)	976 (17.7)
Child ALRI status at baseline	No	2229 (80.8)	2225 (80.9)	4454 (80.9)
	Yes	529 (19.2)	525 (19.1)	1054 (19.1)
Location of cooking quarter	Inside the living house	1008 (36.5)	972 (35.3)	1980 (35.9)
	Separate kitchen	1750 (63.5)	1778 (64.7)	3528 (64.1)
Secondary cookstove type	Traditional stove	2539 (92.1)	2524 (91.8)	5063 (91.9)
	Improved stove	219 (7.9)	226 (8.2)	445 (8.1)
Frequency of “Injera” baking event	One or more per day	383 (13.9)	372 (13.5)	755 (13.7)
	Every other day	303 (11.0)	292 (10.6)	595 (10.8)
	Every 3 days	2014 (73.0)	2035 (74.0)	4049 (73.5)
	Every 4 or more days	58 (2.1)	51 (1.9)	109 (2.0)
Total (%)		2758 (100)	2750 (100)	5508 (100)

### Childhood ALRI follow-up

The first follow-up visit was carried out from September to November 2018; the second took place from December to February 2019; the third occurred from March to May 2019, and the fourth visit was conducted from June to August 2019 at the entire 100 clusters. Then, this trial study was terminated at the planned target of 1 year after the last participant had been randomized. Among the overall follow-up visits of 22,032 in the enrolled population, data were obtained from a total of 19,792 (89.8%) child observations (9860 and 9932 in the intervention and control arms respectively). Among the total of 2240, only 1014 (503 in the control, 511 in the intervention arm) were recorded as new LTF incidence during the entire follow-up period; whereas 1226 (589 in the control and 637 in the intervention arm) were registered as repeated LTF observations in the preceding rounds (*See* Additional file 1).

Among the total data of 19,792 attained; 5313, 5055, 4828, and 4596 data were obtained from the first, second, third, and fourth visits respectively. In addition, there were no entire cluster level losses and exclusions in both arms among the initially enrolled participants. Nevertheless, a total of 2240 (10.2%); (10.4% from the intervention and 9.9% from the control arm) participant visits were LTF after randomization during the entire follow-up period due to moving out of study area, consent withdrawals, child deaths or not at home after a repeated home visit, and reasons for attrition were similar between the intervention and control arms (Additional file 1). Hence, since the reasons for LTF were not related to the given study in both arms, the entire 2240 LTF child observations were omitted from the analysis and the observed 19,792 data can be viewed as a random sample from the population.

Concerning harms, there was 92 episode of childhood cooking-related burn events among the ITT population during the entire follow-up period (41 in the intervention and 51 in the control group); and there was no difference in incidence rates of cooking-related burn events between the intervention and control groups with an incidence rate ratio (IRR) of 0.80 (95% CI: 0.53–1.21) as demonstrated below in Table 2.

**Table 2:** Incidence of cooking-related burn events among the intention-to-treat population

Intervention (*n* = 9860)		Control (*n* = 9932)		IRR (95% CI)
Number of burn events	Incidence rate	Number of burn events	Incidence rate	
41	0.42	51	0.51	0.80 (0.54–1.22)

### Numbers analyzed

Among the enrolled and received the intended treatment participants (5508) in the beginning, follow-up data were obtained from a total of 5333 (96.82%) children (2659 from the intervention and 2674 from the control group) for at least one follow-up visit within 50 clusters in each arm. This establishes the ITT population dataset within 100 clusters in both arms and included in each analysis. However, 175 children were completely omitted from the analysis due to LTF between the baseline and first follow-up visit (91 from the intervention, and 84 from the control arm). For the ITT population, the average cluster size was 53.33 (53.18 for intervention and 53.48 for control arm); and the childhood ALRI prevalence was 19.31% (95% CI: 18.30–20.40) at baseline with 19.18 and 19.45% in the intervention and control arms respectively as depicted in Table 3.

**Table 3:** Characteristics of clusters and participants in the intention-to-treat population

Characteristics	Intervention arm	Control arm	Both arm
Number of clusters	50	50	100
Number of children	2659	2674	5333
Mean cluster size (SD)	53.18 (12.13)	53.48 (12.39)	53.33 (12.2)
CoV for cluster size	0.228	0.232	0.229
Childhood ALRI prevalence rate	19.2	19.5	19.3
Cluster-level variance			2.336E-11
Individual-level variance			3.29
Cluster level ICC			7.10E-12

### Childhood ALRI outcome estimations

**Fig. 2:**
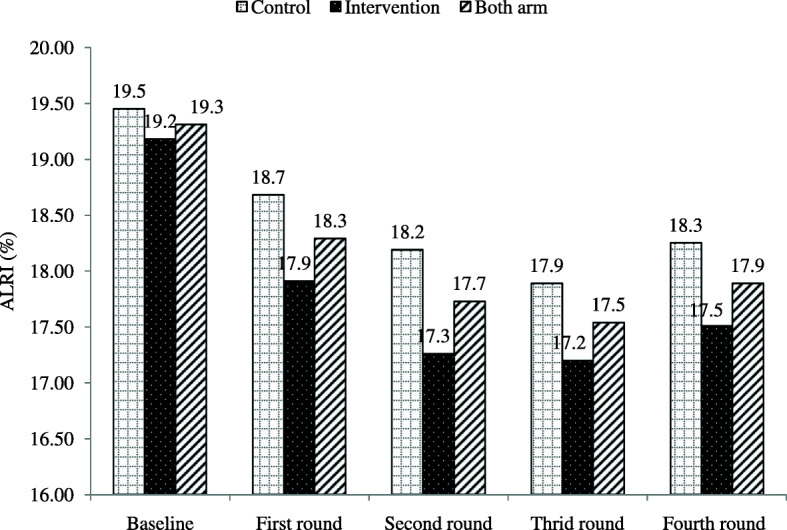
Childhood acute lower respiratory infection rates by round and arm for the trial study entitled effect of improved cookstove intervention on childhood acute lower respiratory infection in Northwest Ethiopia

As to childhood ALRI outcome estimations, there were 3540 episodes of childhood ALRI in the overall ITT population (1732 in the intervention and 1808 in the control group) giving a longitudinal childhood ALRI prevalence rate of 17.9% (95% CI: 17.4–18.4) during the entire follow-up period (17.5 and 18.3% in the intervention and control arm respectively). The childhood ALRI prevalence rate distribution was found to be 18.3% for the first round, 17.7% for the second, 17.5% for the third, and 17.9% for the fourth round as displayed in Fig. 2 by arm.

### Intervention effect estimation

Concerning the predicted effect of the intervention, the IBS intervention was not found to have a statistically significant effect on the longitudinal ALRI among under 5 years old children with an estimated AOR of 0.95 (95% CI: 0.89–1.02) compared with the continuation of an open fire TBS. Nevertheless, as demonstrated by the GEE model in Table 4, the longitudinal change in childhood ALRI was significantly associated with age, baseline childhood ALRI, location of cooking quarter, secondary cookstove type (i.e., cookstove used for other cooking purposes) and frequency of “Injera” baking event measured at baseline.

**Table 4:** Effect of intervention on childhood acute lower respiratory infection

Parameters		AOR (95% CI)	*P-*value
Treatment arm	Intervention	0.95 (0.89,1.02)	0.180
	Control	1	
Gender	Female	1.07 (1.0,1.15)	0.059
	Male	1	
Age of child at entry	< 1 Year old	1	
	1 Year old	0.93 (0.85,1.01)	0.095
	2 Years old	0.71 (0.65,0.78)*	0.001
	3 Years old	0.54 (0.48,0.61)*	0.001
Baseline ALRI	Yes	1.24 (1.14,1.36)*	0.001
	No	1	
Location of cooking quarter	Inside living house	1	
	Separate kitchen	0.78 (0.73,0.83)*	0.001
Secondary cookstove type	Traditional	1	
	Improved	0.62 (0.52,0.74)*	0.001
Frequency of baking event	One or more per day	1	
	Every other day	0.76 (0.67,0.86)*	0.001
	Every 3 days	0.73 (0.66,0.79)*	0.001
	Every 4 or more days	0.65 (0.50,0.86)*	0.002
Visit	1	1	
	2	0.96 (0.87,1.06)	0.430
	3	0.95 (0.85,1.05)	0.324
	4	0.97 (0.88,1.08)	0.619

*significantly associated at ***p-***value < 0.05

## Discussions

This large sample, community-level cluster randomized controlled closed cookstove trial was aimed at determining the effect of IBS intervention on childhood ALRI at the individual child level in Northwest Ethiopia compared with the continuation of open fire TBS. The biomass-fuelled TBS was a major (94.5%) household energy source for baking purpose in the study locality; and the majority (95.8%) of the households practice extra indoor burning events such coffee ceremony, burning incense inside the house as well as outdoor burning events such as burning rubbish and charcoal production was common (38.1%) observable practices in the study area [23].

In this trial, data were obtained from 5333 children (2659 from the intervention and 2674 from the control group) for at least one follow-up visit, and this was used as an ITT population dataset within 100 clusters in both arms. The overall longitudinal childhood ALRI prevalence rate distribution by round was showed a steep reduction followed by a slight increase in similar fashion in both arms (Fig. 2) which might be explained by time variation of the data collection rounds undertaken which is linked with seasonal factors that have a major effect on the magnitude of both ALRI and HAP in Ethiopia [41].

The predicted effect of the biomass-fuelled IBS intervention was not found to have a statistically significant effect on the risk of repeated childhood ALRI response among under 5 years old children in Northwest Ethiopia with an estimated AOR of 0.95 (95% CI: 0.89–1.02) compared with the continuation of an open burning TBS method. This finding mirrors the previous cross-sectional study, conducted as baseline part of this stove trial project, which reported that the odds of childhood ALRI did not show a significant association among children living in households with IBS when compared with children living in households with the TBS type with an estimated AOR of 0.78 (95% CI: 0.52–1.15) [23]. Likewise, a recent policy research working paper reported no evidence that “Mirt” IBS intervention reduced the risk of respiratory symptoms among older children in rural Ethiopia [12].

This finding is also similar to a recent large sample size stove trial in Malawi, which found no evidence that an improved biomass-fuelled cookstove intervention reduced the risk of pneumonia among young children in rural Malawi with an incidence rate ratio of 1·01 (95% CI:0·91, 1·13) with incidence rates of 15·76 (95% CI 14·89–16·63) and15·58 (95% CI 14·72–16·45) per 100 child-years in the intervention and control arms respectively [13]. Our finding is also in agreement with two previous biomass-burning stove intervention studies in Guatemala and Peru, the Guatemala randomized controlled stove trial reported no statistically significant reduction in childhood pneumonia among intervention groups following ICS intervention with a rate ratio of 0·84 (95% CI: 0·63–1.13) [11], and the Peru intervention study found no effect on ALRI symptoms among children under 5 years old following ICS intervention with a rate ratio of 2.45 (95%CI:0.82–7.39) [47].

Our findings do, however; contradict the findings of two previous biomass-burning stove intervention studies in Rwanda and Guatemala which found evidence of health effects among children following biomass-burning ICS interventions [10, 48]. The Rwanda large-scale stove trial reported that a natural draft rocket-style cookstove intervention effectively reduced acute respiratory infection (ARI) prevalence by 25% among children < 5 years with a prevalence ratio of 0.75 (95% CI: 0.60–0.93) [48]. Correspondingly, the field experiment carried out in rural Guatemala reported a 63.3% reduction in respiratory symptoms among children following wood-burning ICS intervention [10].

The possible explanation for the difference between our findings and the findings of previous biomass-burning stove intervention trials might be due to the particular variation of traditional cooking device requirements and trial stove design among others. For instance, unlike our trial stove [21, 22], the Guatemala trial stove reduces smoke exposure mainly by removing emissions to the outdoor environment with a chimney rather than by improving combustion efficiency [10]. In addition, since the effectiveness of cookstoves differs from an ethnic group, geographical location, and local fuel resources [49], the possible explanation for the difference might be the reflection of the difference in geographical location and cultural variations.

Generally, in our trial, the lack of intervention effect on childhood ALRI might be due to the failure of the biomass-fuelled IBS intervention that could not sufficiently reduce HAP to have a preventive health effect; which requires adequate exposure reduction to levels approaching the WHO guideline value to prevent childhood ALRI attributable to HAP exposure [50]. Thus, the most important implication of this finding is that there is inadequate evidence to recommend the biomass-fuelled IBS intervention for the prevention of childhood ALRI compared to the use of open burning TBS.

Consequently, in countries with limited available resources like Ethiopia, an improved stove intervention has to be rigorously proven to be highly effective in reducing the burden of HAP related diseases; and, an effective cooking solution that could potentially reduce smoke exposure sufficiently by removing emissions to the outdoor environment with a chimney rather than by improving combustion efficiency [10] is needed to avert the adverse health effect of HAP, particularly, acute lower respiratory infection among children, who are the most vulnerable and sensitive segment of the population to the damaging health effects of HAP [6, 7].

Finally, even though the IRR was not statistically significant, there was a 20% reduction in the risk of childhood cooking-related burn event in the intervention group, suggesting the IBS intervention might offer a considerable safety advantage over the open TBS method (IRR = 0.80: 95% CI:0.53–1.21). Besides, the IBS intervention would achieve economic and environmental benefits by reducing household fuel consumption and by the direct reduction of the amount of wood burned which can, in turn, decrease environmental destruction and pollution [51].

### Limitations

We acknowledge several limitations in our trial such as the un-blinded nature of the stove intervention; and the lack of intervention effect on childhood ALRI might be explained by exposure to other sources of HAP pollution in the study area, including other cooking activities and presence of extra indoor burning events such as the Ethiopian coffee ceremony with burning incense in the household as well as the presence of outdoor burning events such as charcoal production and rubbish burning near study households that could have diluted any potential effects of the IBS intervention. Also, due to the large sample size accompanied with the lack of adequate resources, we were unable to undertake HAP concentration measurements for every household in each arm.

### Generalisability

This fairly large-sample cookstove trial was completed effectively from a methodological and practical point of view; and our sample was analogous to the wider population of households that uses biomass-fuelled TBS as a major household energy source for cooking purposes throughout Ethiopia, which is typically representative of the great majority (95%) of biomass fuel user households [52] and (80%) traditional stove user population in Ethiopia [53]. Also, this trial study was done in one of the LMICs where ALRI is one of the leading causes of morbidity in children; and most households use biomass fuels for cooking. Therefore, the findings of this trial are generalizable to similar settings in Ethiopia and other low and middle-income countries at an individual child level.

## Conclusions

In conclusion, we found no evidence that an intervention comprising biomass-fuelled IBS reduced the risk of repeated childhood ALRI at the individual child level in Northwest Ethiopia compared with the continuation of an open burning TBS. Therefore, effective cooking solutions that could potentially reduce the negative health impact of HAP are needed to avert the adverse health effect of HAP, particularly, childhood ALRI in the study area. Also, the results of this large-sample trial should have important policy implications in Ethiopia and beyond. Besides, further researches are needed to establish a longer-term health effect of locally made IBS interventions by addressing the limitations of our trial.

## Supplementary Information

**Additional file 1.** Tabular presentation of lost to follow-up events among enrolled participants for the trial study entitled effect of improved cookstove intervention on childhood acute lower respiratory infection in Northwest Ethiopia.

## Data Availability

The data that support the findings of this study are available from the corresponding author on reasonable request and with permission of the “Mecha” Health and Demographic Surveillance research center at Bahir Dar University in Ethiopia.

## Abbreviations

ALRI: Acute Lower Respiratory Infection
AOR: Adjusted Odds Ratio
CI: Confidence Interval
COR: Crude Odds Ratio
CoV: Coefficient of variation
GEE: Generalized Estimation Equation
HAP: Household Air Pollution
IBS: Improved baking Stove
ICC: Intra-cluster Correlation Coefficient
ICS: Improved Cookstove
IMCI: Integrated Management of Childhood Illnesses
IRR: Incidence Rate Ratio
ITT: Intention-to-Treat
LMICs: Low-and Middle-Income Countries
LTF: Lost to Follow-up
MHDSS: Mecha Health and Demographic Surveillance System
TBS: Traditional Baking Stove
WCS: Working Correlation Structure
WHO: World Health Organization
