# Supplementary material for: Effect of improved cookstove intervention on childhood acute lower respiratory infection in Northwest Ethiopia: a cluster-randomized controlled trial
Source: BMC Pediatr. 2021 Jan 4;21:4. doi: 10.1186/s12887-020-02459-1 (PMC7780395; doi:10.1186/s12887-020-02459-1)
Supplement: Supplementary file 1 — Additional file 1. Tabular presentation of lost to follow-up events among enrolled participants for the trial study entitled effect of improved cookstove intervention on childhood acute lower respiratory infection in Northwest Ethiopia. [file 12887_2020_2459_MOESM1_ESM.pdf]

Characteristics		Treatment arm		Total (%)	X^2 *(P-value)*
		Control (%)	Intervention (%)		
Lost to follow-up	Yes	1,092 (9.9)	1,148 (10.4)	2,240 (10.2)	1.65 (0.199)
	No	9,932 (90.1)	9,860 (89.6)	19,792 (89.8)	
	Total	11,024 (100)	11,008 (100)	22,032 (100)	
Reasons for lost to follow-up	Moved out of study area	558 (51.1)	549 (47.8)	1,107 (49.4)	3.94 (0.268)
	Consent withdrawal	318 (29.1)	377 (32.8)	695 (31.0)	
	Death of child	148 (13.6)	148 (12.9)	296 (13.2)	
	Not at home	68 (6.2)	74 (6.5)	142 (6.4)	
	Total	1,092 (100)	1,148 (100)	2,240 (100)	
